# Whole-genome single nucleotide polymorphism analysis for typing the pandemic pathogen *Fusarium graminearum* sensu stricto

**DOI:** 10.3389/fmicb.2022.885978

**Published:** 2022-07-18

**Authors:** Tomasz Kulik, Tomasz Molcan, Grzegorz Fiedorowicz, Anne van Diepeningen, Alexander Stakheev, Kinga Treder, Jacek Olszewski, Katarzyna Bilska, Marco Beyer, Matias Pasquali, Sebastian Stenglein

**Affiliations:** ^1^Department of Botany and Nature Protection, University of Warmia and Mazury in Olsztyn, Olsztyn, Poland; ^2^Department of Bioinformatics, Institute of Biochemistry and Biophysics, Polish Academy of Sciences (PAN), Warsaw, Poland; ^3^Biointeractions and Plant Health, Wageningen Plant Research, Wageningen, Netherlands; ^4^Shemyakin and Ovchinnikov Institute of Bioorganic Chemistry, Russian Academy of Sciences, Moscow, Russia; ^5^Department of Agriculture Systems, University of Warmia and Mazury in Olsztyn, Olsztyn, Poland; ^6^Experimental Education Unit, Olsztyn, Poland; ^7^Agro-Environmental Systems, Environmental Monitoring and Sensing Unit, Department of Environmental Research and Innovation, Luxembourg Institute of Science and Technology, Esch-sur-Alzette, Luxembourg; ^8^Department of Food, Environmental and Nutritional Sciences, University of Milan, Milan, Italy; ^9^National Scientific and Technical Research Council, Godoy Cruz, Argentina; ^10^Universidad Nacional del Centro de la Provincia de Buenos Aires, Tandil, Argentina

**Keywords:** *Fusarium graminearum* sensu stricto, *F. graminearum* complex, whole-genome sequencing, plant pathogen, identification

## Abstract

Recent improvements in microbiology and molecular epidemiology were largely stimulated by whole- genome sequencing (WGS), which provides an unprecedented resolution in discriminating highly related genetic backgrounds. WGS is becoming the method of choice in epidemiology of fungal diseases, but its application is still in a pioneer stage, mainly due to the limited number of available genomes. Fungal pathogens often belong to complexes composed of numerous cryptic species. Detecting cryptic diversity is fundamental to understand the dynamics and the evolutionary relationships underlying disease outbreaks. In this study, we explore the value of whole-genome SNP analyses in identification of the pandemic pathogen *Fusarium graminearum sensu stricto* (*F.g*.). This species is responsible for cereal diseases and negatively impacts grain production worldwide. The fungus belongs to the monophyletic fungal complex referred to as *F. graminearum* species complex including at least sixteen cryptic species, a few among them may be involved in cereal diseases in certain agricultural areas. We analyzed WGS data from a collection of 99 *F.g.* strains and 33 strains representing all known cryptic species belonging to the FGSC complex. As a first step, we performed a phylogenomic analysis to reveal species-specific clustering. A RAxML maximum likelihood tree grouped all analyzed strains of *F.g.* into a single clade, supporting the clustering-based identification approach. Although, phylogenetic reconstructions are essential in detecting cryptic species, a phylogenomic tree does not fulfill the criteria for rapid and cost-effective approach for identification of fungi, due to the time-consuming nature of the analysis. As an alternative, analysis of WGS information by mapping sequence data from individual strains against reference genomes may provide useful markers for the rapid identification of fungi. We provide a robust framework for typing *F.g.* through the web-based PhaME workflow available at EDGE bioinformatics. The method was validated through multiple comparisons of assembly genomes to *F.g.* reference strain PH-1. We showed that the difference between intra- and interspecies variability was at least two times higher than intraspecific variation facilitating successful typing of *F.g*. This is the first study which employs WGS data for typing plant pathogenic fusaria.

## Introduction

Fungi are among the most diverse groups of eukaryotic organisms on earth and include a wide range of taxonomic groups with various morphological and phenotypic characters, ecologies and life cycles (Hyde et al., 2019; Naranjo-Ortiz and Gabaldón, 2020). With their complex and often cryptic nature, fungi are widespread from polar to tropical habitats acting as decomposers of organic matter and symbionts of algae and plants (Frac et al., 2018; Bonfante, 2019). Mushrooms are used to make foods rich in mycoproteins, dietary fibers, vitamins and antioxidants (Valverde et al., 2015; Ulian et al., 2020). Fungi are also well known as producers of a vast array of secondary metabolites reflecting their adaptation to a diversity of environments (Boruta, 2018; Tralamazza et al., 2019). Besides their beneficial roles for food production, medicine and other industries, fungi can be harmful to the health of humans and animals as well as for the environment (Wu and Hao, 2019). Around 625 fungi were found to affect vertebrates, and a further 700 species could be associated with humans, either as commensals, microbiome community members or as pathogens (Fisher et al., 2020; de Hoog et al., 2021). From about 100.000 known species, around 20% are involved in 70–80% of plant diseases (Persley, 1993; Ray et al., 2017). Besides, many fungi are responsible for significant qualitative loss of food and feed by mycotoxin contamination (Marin et al., 2013; Ukwuru et al., 2017), which has negative effects on humans and animals (Zain, 2011).

Undoubtedly, taxonomic information is crucial for understanding fungal diversity and evolution. Taxonomy provides a common language for scientists and the regulatory community (Raja et al., 2017a,b), and is especially critical in clinical, environmental, food and biological applications (Kulik et al., 2020). Classical taxonomic methods enable the determination of the species based on morphological characteristics. However, laborious culture-based methods are often neglected by scientists mostly due to morphological/phenotypic variability and pleomorphism (Capote et al., 2012; Sharma and Sharma, 2016). The field of mycology has come to accept DNA-based technology, which has long been used for identification purposes. The most prevalent approaches are based on polymerase chain reaction (PCR), which targets specific DNA regions allowing species determination. Numerous molecular approaches have been developed to detect races, formae speciales, mating types and mycotoxin genotypes among different fungal taxa. However, the necessity for multiple laboratory tests for fungi remains laborious, expensive, and time-consuming (Kulik et al., 2020).

More universal DNA barcoding promises to be a valuable approach for high-throughput identification of fungi. Defining species through DNA barcoding involves sequence comparison of short and unique DNA barcodes (ca. 400–800 bp) which are amplified and sequenced with the use of universal primers. Today, the internal transcribed spacer (ITS) region in the ribosomal RNA gene cluster has been validated as the best DNA barcode marker for fungi. However, its reliability as taxonomic marker for fungi has been criticized, because of: (i) the presence of divergent intragenomic ITS sequences in several fungal lineages (Kiss, 2012) and (ii) lack of sufficient sequence polymorphism facilitating recognition of some closely related species (Seifert, 2009; Kiss, 2012). For many phytopathogenic genera, e.g., *Alternaria, Botryosphaeria, Cercospora, Diaporthe*, and *Fusarium* ITS sequence alone cannot be used to identify most species (Seifert, 2009; Sharma et al., 2015; Kashyap et al., 2017).

A number of fungi previously proved to be a single species proved species complexes composed of multiple cryptic species. Identification of these diverged species is usually achieved through multilocus sequence typing (MLST). This approach involves the amplification of housekeeping genes by PCR, followed by DNA sequencing of amplicons and defining an allelic profile corresponding to a Sequence Type (ST) (Taylor and Fisher, 2003). The use of MLST allows us to detect the emergence of novel genotypes or sequence variants that can be additionally stored in a publicly accessible database. The obvious limitation of MLST can be linked to its failure to fully identify genomic diversity. Analysis of a limited number of loci in the genome is often not discriminative enough to be useful for outbreak detection (Teatero et al., 2015; Uelze et al., 2020). In addition, MLST analysis of various fungal complexes appears to be more complicated than bacterial pathogens because of the less conserved MLST loci which require multiple genus- or even species complex-specific primers for successful amplification. The choice of suitable primer sets is thereby critical and often dependent on the ITS sequence (Wickes and Romanelli, 2020).

Recent advances in genomic sequence analysis and Single Nucleotide Polymorphism (SNP) discovery have empowered researchers to explore microbial diversity in more detail. The increasing amount of sequenced fungal genomes opens up the possibility to better understand the tremendous diversity of fungi and their evolution. Fungal genomes are relatively small in comparison to plant and animal genomes, but vary from several to nearly 1,000 megabases (Mb) (Stajich, 2017). Genomes of fungi are dynamic in nature and rapid progress in high throughput sequencing methods pinpoint to different mechanisms of genome evolution (Mohanta and Bae, 2015; Priest et al., 2020). Among them gene duplication, polyploidy, chromosomal rearrangements, interspecific hybridization, introgression and horizontal gene transfer are considered to be the main mechanisms shaping genetic diversity of fungi (Albertin and Marullo, 2012; Priest et al., 2020). In addition, fungal genomics demonstrate significant divergence between fungal genomes that can be linked to host/niche specialization and lifestyles, and allows the research community to gain insight into the fundamental aspects of fungal biology (Gladieux et al., 2014). Nowadays, the rapid generation of genomic data contributes significantly to our understanding of fungal diseases, fungicide resistance, toxicology, and to the discovery of biosynthetic gene clusters that underlie the capacity to produce secondary metabolites (Tralamazza et al., 2019; Pasquali et al., 2020; Rampersad, 2020). The whole-genome strategies allow for determination of fungal species, support fungal taxonomy and dispersal patterns (Araujo, 2014; Araujo and Sampaio-Maia, 2018). Among these approaches, genome-wide SNPs provide increased resolution to evaluate fungal diversity (Araujo, 2014; Araujo and Sampaio-Maia, 2018). SNPs display relatively low mutation rates and are evolutionarily stable (Leekitcharoenphon et al., 2012), making them ideal to establish divergence between species as well as strains (Schork et al., 2000; Filliol et al., 2006; Shakya et al., 2020). Usually, SNPs are identified by mapping sequence data from individual strains against a closely related reference genome. Such analysis is based on a set of generated core SNPs, which is covered by all studied genomes including reference genome. SNP distance matrices determined from combinations of pairwise SNP distances enable further phylogenetic analysis (Uelze et al., 2020). It is worth noting, however, that the application of genome-based approaches in mycology is still in a pioneer stage. Validation of diagnostic tools requires the incorporation of genomic data covering a large fraction of cryptic diversity within the groups of morphologically indistinguishable species. Unfortunately, many fungal complexes still lack sufficient numbers of genomes to fully explore their efficiency for diagnostic purposes.

Our previous large scale sequencing project was launched in order to obtain genome sequences from a large collection of strains of the pandemic pathogen *Fusarium graminearum sensu stricto* (*F.g.*) (Wyrebek et al., 2021). This cryptic species is mainly responsible for two cereal diseases: Fusarium Head Blight (FHB) of wheat and barley and Fusarium Ear Rot (FER) of maize. Both diseases have led to major economic losses for the cereal-based feed and food supply chains worldwide (van der Lee et al., 2015). *F.g*. belongs to the *F. graminearum* species complex (FGSC), which includes at least sixteen cryptic species (Sarver et al., 2011), among them a few may be involved in cereal diseases in certain agricultural areas (van der Lee et al., 2015). Determination of cryptic diversity within field populations enables us to better understand their dynamics and the evolutionary relationships underlying disease outbreaks (van der Lee et al., 2015; Wyrebek et al., 2021). We have previously explored the value of mitochondrial sequences for diagnostic purposes of *F.g.* We showed that mitochondrial-based SNP analysis are useful for typing most, but not all, strains of *F.g.* We also underlined the limitations of clustering-based identification approaches for FGSC species using mitochondrial sequences (Wyrebek et al., 2021).

In this paper, sequence data from previous WGS project were first used to assemble genomes from a total of 99 *F.g.* strains and 33 strains representing all known cryptic species from the FGSC complex. We constructed a phylogenomic tree to demonstrate species-specific clustering for species recognition. We also show that the web-based calculation of the number of SNPs *via* comparison to reference PH-1 strain of *F.g.* provides an easy method of typing *F.g*. This is the first report on typing plant pathogenic fusaria through WGS data.

## Materials and methods

### Fungal strains and genome assembly

In total, 136 strains were analyzed in this study, with 99 *F.g.* strains and 33 strains representing all known cryptic species from the FGSC complex: *Fusarium acaciae-mearnsii* (three strains), *Fusarium aethiopicum* (one strain), *Fusarium asiaticum* (*n* = 3), *Fusarium austroamericanum* (*n* = 2), *Fusarium boothii* (*n* = 4), *Fusarium brasilicum* (*n* = 1), *Fusarium cortadariae* (*n* = 3), *Fusarium gerlachii* (*n* = 2), *Fusarium louisianense* (*n* = 2), *Fusarium meridionale* (*n* = 2), *Fusarium mesoamericanum* (*n* = 1), *Fusarium nepalense* (*n* = 2), *Fusarium ussurianum* (*n* = 4), *Fusarium vorosii* (*n* = 2), and strain CBS 123663 (NRRL34461), which lacks a Latin binomial. In addition, four single strains from the closely related morphospecies *F. culmorum*, *F. pseudograminearum*, *F. sambucinum* and *F. venenatum* were incorporated into SNP analysis. Detailed information on all strains used in this study are included in Supplementary File 1.

### Whole-genome sequencing and genome assembly

For the majority of strains, whole fungal genomic DNA was sequenced by Macrogen (Seoul, South Korea). Libraries were prepared using the KAPA HyperPlus Kit (Roche Sequencing Solutions, Pleasanton, CA, United States). An Illumina HiSeq X Ten was used to sequence the genomes using a paired-end read length of 2 × 150 bp with an insert size of 350 bp. For 12 strains: ar1 (CBS_139514), ar3 (119-12), us2 (CBS_119173), po12 (CBS138561), po13 (CBS138562), po14 (CBS138563, 0357), fas1 (CBS110258), fam3 (CBS123662), fau1 (CBS110244), fbo3 (CBS119170), fme2 (CBS110260) and sa2 (CBS119800), whole genome libraries were prepared using a Nextera XT kit (Illumina, San Diego, CA, United States) and sequenced on the Illumina Miseq platform with the 250 bp paired-end read, version 2. The sequencing quality was assessed *via* FastQC (ver. 0.11.9) (Andrews, 2010). Low-quality reads were trimmed using Trimmomatic (v.0.36) (Bolger et al., 2014) and the genomes were assembled *via* SPAdes (v.3.13.2) (Nurk et al., 2013) with k-mer values of 21, 33, 55, 77, 99, 127 and using the “careful” option to reduce mismatches. Substitution errors are typical in Illumina reads at the rate of 0.02–0.05%. Automated correction of sequencing errors was performed by Spades, which incorporates the BayesHammer error correction tool propagating corrections in *k*-mers to corrections in reads (Nikolenko et al., 2013). The completeness of the assembly was evaluated using BUSCO (Simão et al., 2015). Genome statistics including genome length and N50 values were calculated using QUAST v. 5.0.2 (Mikheenko et al., 2018). The project was submitted to NCBI BioProject under accession: PRJNA677929. GenBank accession numbers are listed in Supplementary File 1. Genomes assembly of eight strains: GCA_900044135.1 (us1, PH-1), GCA_000966635.1 (ca1, 233423), GCA_001717915.1 (ca2, DAOM180378), GCA_900073075.1 (bra1, CML3066), GCA_012959185.1 (is1, TaB10), GCA_000599445.1 (sy1, CS3005), GCA_001717845.1 (fas2, NRRL 6101) and GCA_001717835.1 (fas3, NRRL 28720) were retrieved from GenBank.

### Phylogenomic analysis

Locally installed PhaME (Shakya et al., 2020) was used to reconstruct phylogeny of the strains from FGSC. The phylogenomic tree was inferred using the RAxML maximum likelihood method (Stamatakis, 2014). The *F. pseudograminearum* strain CS3096 was used as an outgroup.

### Single nucleotide polymorphism analysis

To extract core SNPs, we used the whole-genome SNP-based phylogeny tool PhaME, which is integrated into the EDGE bioinformatics platform^1^ (Li et al., 2017). PhaME extracts SNPs from the core genome identified through comparison of assembly genome to the reference PH-1 strain of *F.g.* (GenBank assembly accession: GCA_900044135.1). The total number of SNPs is counted as the number of positions that are variable between two genomes.

### Phylogenetic analysis

A set of 39 single-copy orthologous genes recommended by the Fungal Tree of Life (Zhang et al., 2020) were subjected for phylogenetic analysis. Orthologous sequences were retrieved from genome assemblies based on homology search for the corresponding nucleotide sequences from the reference genome of PH-1 using Geneious Prime software (Biomatters Ltd., New Zealand). The sequences were individually aligned using MAFFT software (v.7.453; Katoh and Toh, 2010) with default settings. Insertions/deletions (INDELs) were excluded from the subsequent data analyses. The best partition schemes and corresponding substitution models were estimated using jModelTest (Minh et al., 2020). Afterward, based on the alignment and obtained models, maximum likelihood analysis was conducted using IQ-TREE 2.0.3 with 1,000 ultrafast bootstrap (Minh et al., 2020). Sequence data from *F. culmorum* (GenBank assembly accession: GCA_019055245.1) and *F. cerealis* (GenBank assembly accession: GCA_019055205.1) was used as an outgroup. Variation within each sequence was identified as a SNP and counted with the use of an in-house Python script. Nucleotide diversity values (π) for were calculated with TASSEL software (v.5.2.40; Bradbury et al., 2007).

## Results and discussion

### Genomic sampling

We analyzed genomes of 99 *F.g.* strains and 33 strains representing all known cryptic species from the FGSC complex. Majority (*n* = 124) of whole-genome sequences were generated in this study. Details of genome assembly quality is given in Supplementary File 1. For genomes sequenced on HiSeq X Ten platform, N50 values ranged from 212 to 6,717 kb and 98.9–100% completeness according to BUSCO. N50 values of genomes sequenced on Miseq ranged from 23 to 176 kb and 91.7–99.9% completeness according to BUSCO. The genome sizes within the FGSC ranged from 34.7 to 37.4 Mb.

### A phylogenomic approach facilitates clustering-based identification of *F.g.*

The pattern of clustering observed on the phylogenomic tree (Supplementary Image 1) sheds new light on the phylogeographic structure and genetic relationship among the species, which plays a significant role in their identification. Species-specific clustering was evident in case of all species represented by more than one strain (Supplementary Image 1). In general, two major sister clades were resolved. The first clade occupied the basal position of the tree and grouped all species from Africa (*F. aethiopicum, F. acaciae-mearnsii*), Asia (*F. asiaticum*, *F. nepalense*, *F. ussurianum* and *F. vorosii*) and Australia (*F. acaciae-mearnsii).* The second larger clade was more diverse and included species endemic to North (*F. gerlachii, F. louisianense*) and South America (*F. austroamericanum, F. mesoamericanum, F. brasilicum*) and New Zealand (*F. cortadariae*). *F. boothii* and *F. meridionale* reported from diverse regions of Asia, Africa, and Latin America (van der Lee et al., 2015) were also clustered in this large clade.

To minimize the impact of geographic variation that may interfere with the results, we incorporated a large set of geographically diverse strains. Such a strategy is especially critical for pandemic species, which are often subdivided into genetically distinct populations (Shakya et al., 2021). *F. graminearum* formed a peripheral clade on the tree separating the species into thirteen major subclades named I to XIII. We observed geographic overlap in these subclades. Five out of nine strains originating from either North or South America were members of subclade I (*n* = 9). Its basal position in the *F.g*. clade may suggest that subclade I includes genotypes with the highest genetic relationship to ancestors of *F.g*. Interestingly, subclade I included the oldest known strains of *F.g.*: un1 (CBS 185.32) and un2 (CBS 104.09), which were isolated/deposited in the collection of the Westerdijk Fungal Biodiversity Institute in 1932 and 1904, respectively. Unfortunately, associated metadata of these two strains do not include information on their geographic origin. The first three subclades (II, III and IV) diverging from subclade I were represented by single strains, among which two: ar4 (114-2) and ar1 (CBS_139514) come from South America and one (sy1, CS3005) from Australia. Most European strains were grouped into clusters VI-XIII. Interestingly, subclades VI (*n* = 7), VII (*n* = 1), VIII (*n* = 10) and IX (*n* = 2) grouped mostly Polish strains. A small subclade X clustered only two strains: ne5 (79E1) originating from Netherlands and po11 (16-390-z) originating from Poland. This small clade was located between Polish subclade IX and west European subclade XI grouping strains (*n* = 34) mostly from West Europe. The observed high degree of geographic clustering of the strains was not observed in subclade XIII (*n* = 27), which diverged from west European subclade XI. It grouped strains from diverse geographic locations such Argentina, Brazil, Italy, Germany, the Netherland, Poland, Russia and Serbia, pointing out evidence of their most recent spread to new geographic locations. Geographic expansion in accessible directions may be also indicated by clustering two European strains ge3 (CS10007) (from Germany) and ru13 (70725) (from Russia) into subclade I. Other examples come from subclade XI, which included a single strain from South Africa (sa1, CBS 119799), and subclade VI, which included strain ir1 (CBS 110263) from Iran.

Close geographic co-occurrence of strains from different subclades might raise important questions regarding their epidemiologic importance, especially if different subclades represent recently introduced genotypes. Transmission of novel strains may drive changes in local population structures with replacement or displacement of the existing genotypes or the creation of a wide variety of admixture genotypes, more than what one could expect from recombination and gene flow among native populations or even endemic species. Dramatic shifts within European field populations of F.g. have been recently reported and mainly concerned the emergence of 15ADON genotypes of *F.g*., which displaced of *F. culmorum*, which had been the major FHB agent of wheat since the 1840s (van der Lee et al., 2015). Also, local temporary shifts of F.g. chemotypes were reported in Luxembourg due to drought effects (Beyer et al., 2014). The most recent survey from Poland highlighted changes in population structure of *F.g.* by replacement of previously reported nivalenol genotypes of *F.g*. by 15ADON genotypes (Bilska et al., 2018). However, another study by Talas and McDonald (2015) showed that German field populations of *F.g*. showed high genetic diversity and limited differentiation among populations supporting the hypothesis of frequent recombination among European *F.g*. strains. A central goal of our further studies is to understand how genetic diversity is structured and maintained within European *F.g*. populations. One of the most urgent tasks will be to determine individual populations within the studied set of strains and to assess admixture. We hypothesize that certain subclades on the phylogenomic tree may include admixture strains with different ancestries. Determination of the level of admixture within European F.g. would clarify the distribution of genetic diversity and population connectivity through gene flow. Recombination enhances genetic diversity, which may increase fitness and facilitate adaptation of pathogens to changing environments.

### A phylogenomic approach enables the detection and clarification of the incorrect taxonomic status of historical strains held in fungal collections

Among the *F.g*. strains, one strain (sa2, CBS 119800) unexpectedly clustered outside the *F.g*. clade and was grouped in the *F. boothii* clade. This strain is held in the fungal collection Westerdijk Fungal Biodiversity Institute (Utrecht, Netherlands) as *F. graminearum* and according to available metadata it was isolated from maize in South Africa where *F. boothii* has been frequently reported (van der Lee et al., 2015). However, our previous mitochondrial-based comprehensive studies did not indicated incorrect taxonomic status of this strain (Kulik et al., 2015; Brankovics et al., 2018; Wyrebek et al., 2021). The complete mitogenome of CBS 119800 (NCBI accession no. KP966554) displays 100% sequence identity to *F.g.* strain CBS 104.09 (NCBI accession no. KR011238). Partial (659 nt) tef gene sequence of CBS 119800 (NCBI accession no. KT855180) shows 100% identity to *F.g*. (NCBI accession no. KP267345), indicating that *tef* alone could not definitively confirm its taxonomic affiliation. Blast search against NCBI non-redundant (nr) nucleotide database with an e-value cutoff of ≤ e-0.0, 100% identity and 100% coverage also yielded nearly 30 hits to *F. boothii.* To further clarify its taxonomic status, we retrieved the complete sequence of the topoisomerase 1 (*top1*) and phosphoglycerate kinase (*pgk*) genes, which have become widely used taxonomic markers for Fusaria (Stielow et al., 2015). Blast searching with the *top1* gene as a query (e-value cutoff of ≤ e-0.0 and 100% coverage) yielded two hits to *F. boothii* (NCBI accession no. KY952952 and KY952951) with 100% sequence identity. However, blast searching with the *pgk* gene as a query did not produce hits with 100% sequence identity, presumably due to the lack of *pgk* sequences from *F. boothii* in GenBank database. To determine sequence similarity of *pgk* between CBS 119800 and other strains of *F. boothii*, we retrieved its sequence from genome assembly of three strains of *F. boothii*: CBS 316.73, CBS 110251 and CBS 119170. Subsequent sequence comparison (data not shown) revealed that all four strains shared 100% identity in the *pgk* gene, thus, supporting its identity as *F. boothii*.

Our results of phylogenomic analysis may also be helpful in resolving the uncertain taxonomic status of CBS 110260. This strain was isolated from maize in Nepal and has been assigned to either *F. asiaticum* or *F. meridionale* or a hybrid strain (O’Donnell et al., 2000, 2004; Ward et al., 2002; Starkey et al., 2007; Yang et al., 2008). Most recent studies by Walkowiak et al. (2016) based on analyses of SNPs and indels suggested that this strain shows 99% sequence identity to *F. meridionale*. Indeed, our phylogenomic approach grouped CBS 110260 together with the strain CBS 110249 (fme1, Supplementary Image 1), which supports its taxonomic assignment as *F. meridionale*. Moreover, positioning of *F. meridionale* on the second large clade suggests that this cryptic species is more closely related to especially *F. cortadariae, F. austroamericanum, F. brasilicum* and even to *F.g.* than to *F. asiaticum*. Additional whole-genome SNP analyses (Table 1) confirmed the above findings. Notably, the number of SNPs (126.189) between CBS 110260 and *F. meridionale* strain (CBS 110249) is in range of intraspecific variability found for *F.g*. (discussed in later sections), which is indicative of its taxonomic assignment as *F. meridionale.*

**TABLE 1:** Results of whole-genome SNP analyses by mapping of assembly genomes to reference CBS 110260 strain.

Assembly genomes		*F. meridionale* CBS 110249	*F. cortadariae* CBS 119183	*F. austroamericanum* CBS 110244	*F. brasilicum* CBS 119179	*F. graminearum* PH-1	*F. asiaticum* CBS 110258
CBS 110260	SNPs	126,189	336,338	335,339	342,238	567,931	577,489
	Linear coverage	98.5%	97.4%	97.4%	97.1%	95.6%	93.5%

### Assessment of genome-wide SNP counts allow successful identification of *F.g.*

One of the major drawbacks of clustering-based strategies for microbial identification is that they are time consuming, require bioinformatics skills, and specialized software and equipment. The remedy for these limitations is intuitive, user-friendly web-based platforms enabling fast and easy processing of next generation sequencing data through numerous cutting-edge tools. We used the PhaME workflow available at EDGE bioinformatics enabling fast counting of the total number of SNPs for determination of *F.g..* The method was validated through multiple comparisons of assembly genomes to reference PH-1 strain. We estimated intra and interspecific differences in the number of SNPs facilitating species recognition (Supplementary File 2). Intraspecific variation calculated *via* comparisons of *F.g.* strains ranged from around 86.000 to nearly 158.000 SNPs, with one exception. The exceptional result was found for the strain CBS 119173, which yielded nearly 209.000 variable SNPs. However, the increased variability of CBS 119173 is not unexpected. CBS 119173 belongs to Gulf Coast population of *F.g.* with higher divergence, as evaluated *via* previous phylogenetic analyses of multilocus DNA sequence data (Starkey et al., 2007). The increased genetic divergence of CBS 119173 was also depicted on phylogenomic tree (Supplementary Image 1) by clustering of this strain separately from the remaining *F.g.* strains.

For cryptic species from the FGSC complex, interspecific variation ranged from 304.164 to 706.454 SNPs being from at least, nearly two times higher than intraspecific variation. Unsurprisingly, higher intraspecific variation was found for closely related morphospecies *F. culmorum*, *F. pseudograminearum*, *F. sambucinum* and *F. venenatum*, and ranged from 1,032,686 to 1,955,620 SNPs being more than 6.5 times higher than intraspecific variation.

### Phylogenetic analyses

Phylogenetic analyses of different housekeeping genes (Supplementary File 3) help to resolve taxonomic relationships in the FGSC (O’Donnell et al., 2000, 2004), but the rather conserved nature of phylogenetic markers might make them unsuitable for exploring variation at the strain level. However, we explored how clustering of strains in phylogenomic tree corresponds with phylogenetic relationships. As expected, the generated maximum-likelihood tree (Supplementary Image 2) failed to support *F.g*. subclades. Most of them were weakly supported (bootstrap scores < 70), with one major exception. In subclade I grouping most of the strains from either North or South America had high bootstrap support (92). In addition, phylogenetic tree supported higher genetic divergence of CBS 119173 (us2) by clustering of this strain at the basal position of the *F.g.* clade. Grouping of cryptic species of FGSC corresponded to phylogenomic clustering and confirmed clarification of taxonomic issues of CBS 119800 (sa2) and CBS 110260 (fme2) as *F. boothii* and *F. meridionale*, respectively.

## Conclusion

Calculation of whole-genome SNP variation allows to determine strains of *Fusarium graminearum* sensu stricto (*F.g*.) based on intra- and interspecific differences in the number of SNPs. Analysis of genome comparison does not require specialized software and can be rapidly performed in a user-friendly web-based workflows. Our results seem to be sufficiently robust to the effects that the quality of the assembly can have on SNPs calling (Olson et al., 2015).

## Data availability statement

The datasets presented in this study can be found in online repositories. The names of the repository/repositories and accession number(s) can be found in the article/Supplementary Material.

## Author contributions

TK conceived the idea, acquired funding, designed the study, and prepared the draft manuscript. TM reconstructed phylogeny of the strains. GF performed assessment of genome-wide SNP counts. AD, AS, JO, KB, MB, MP, and SS provided fungal strains for analysis. KT, KB, and GF provided the technical support during the study. All authors read the manuscript, critically revised, agreed to the content of the manuscript, read and approved the final manuscript.

## Conflict of interest

The authors declare that the research was conducted in the absence of any commercial or financial relationships that could be construed as a potential conflict of interest.

## Publisher’s note

All claims expressed in this article are solely those of the authors and do not necessarily represent those of their affiliated organizations, or those of the publisher, the editors and the reviewers. Any product that may be evaluated in this article, or claim that may be made by its manufacturer, is not guaranteed or endorsed by the publisher.

## Footnotes

1
https://edgebioinformatics.org/

## Abbreviations

F.g: *Fusarium graminearum* sensu stricto
FGSC: *Fusarium graminearum* complex
WGS: whole-genome sequencing
SNP: single nucleotide polymorphism
ITS: internal transcribed spacer
