# Supplementary material for: Whole-genome single nucleotide polymorphism analysis for typing the pandemic pathogen Fusarium graminearum sensu stricto
Source: Front Microbiol. 2022 Jul 18;13:885978. doi: 10.3389/fmicb.2022.885978 (PMC9339996; doi:10.3389/fmicb.2022.885978)
Supplement: Supplementary file 4 [file Image_1.pdf]

Supplementary Image 1. The phylogenomic tree resulting from the RAxML Maximum Likelihood showing branches with bootstrap support values > 70%. The *F. pseudograminearum* strain CS3096 was used as an outgroup.

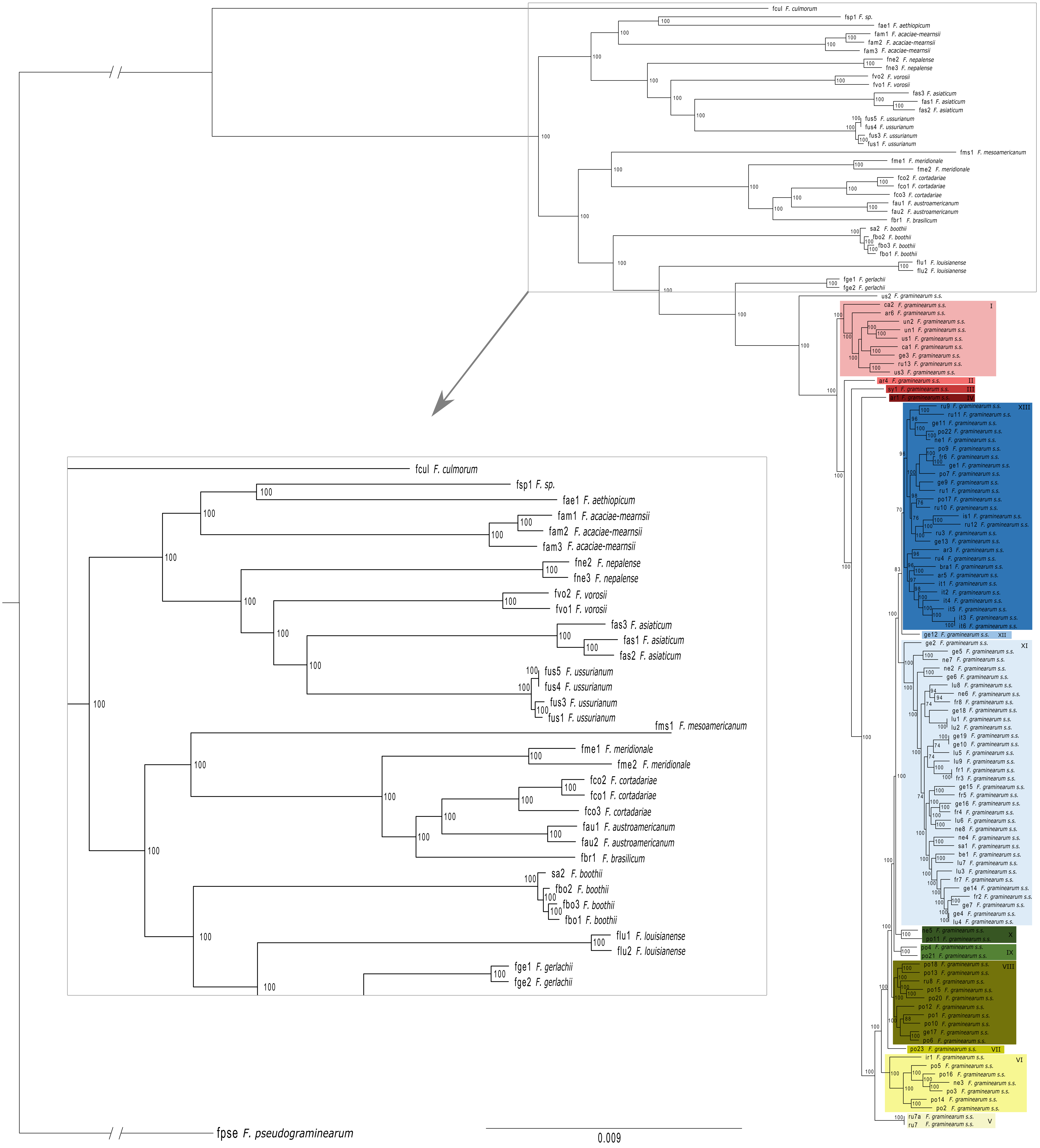

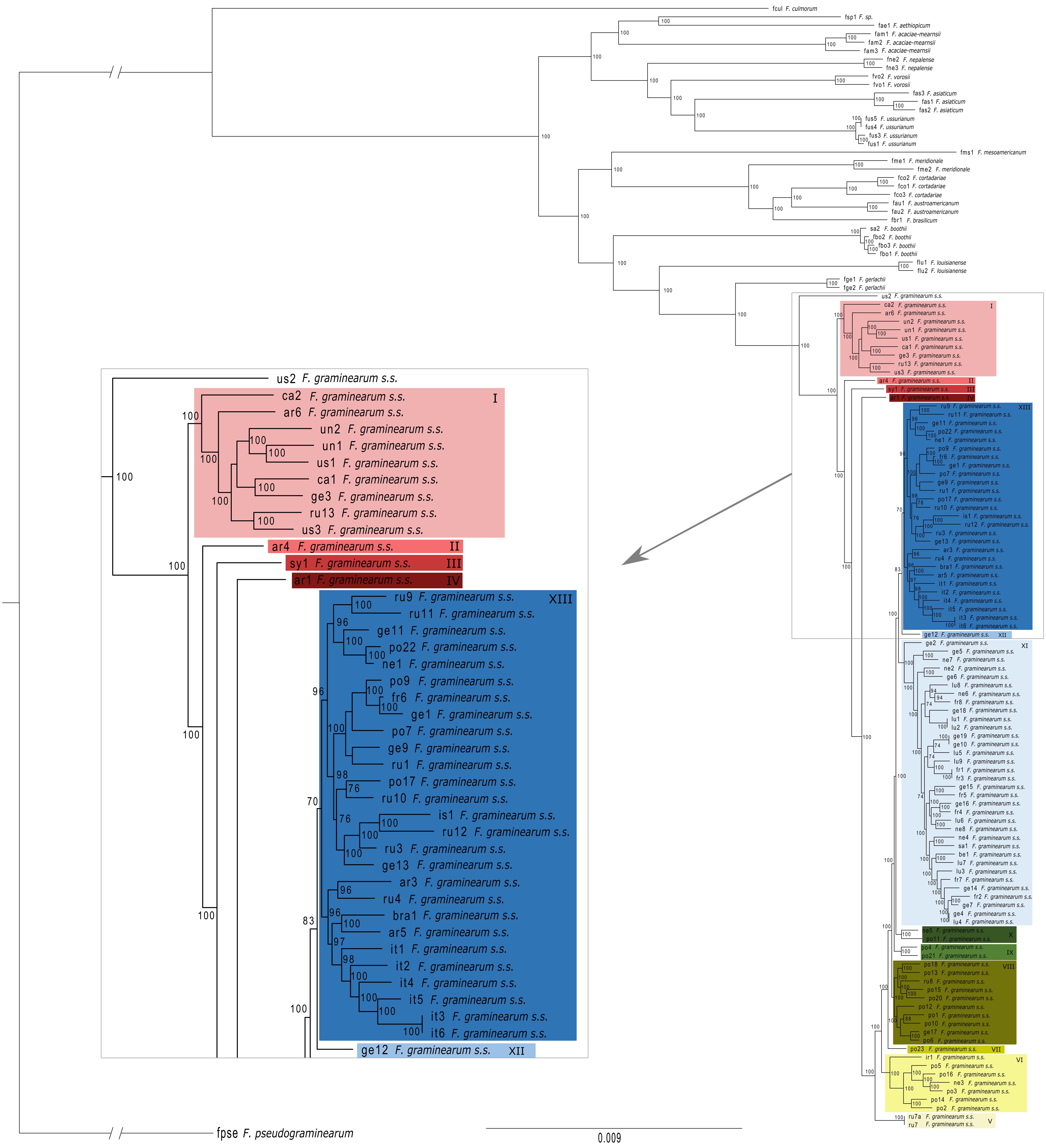

fcul F. culmorum
fsp1 F. sp.
fae1 F. aethiopicum
fam1 F. acaciae-mearnsii
fam2 F. acaciae-mearnsii
fam3 F. acaciae-mearnsii
fne2 F. nepalense
fne3 F. nepalense
fvo2 F. vorosii
fvo1 F. vorosii
fas3 F. asiaticum
fas1 F. asiaticum
fas2 F. asiaticum
fus5 F. ussurianum
fus4 F. ussurianum
fus3 F. ussurianum
fus1 F. ussurianum
fms1 F. mesoamericanum
fme1 F. meridionale
fme2 F. meridionale
fco2 F. cortadariae
fco1 F. cortadariae
fco3 F. cortadariae
fau1 F. austroamericanum
fau2 F. austroamericanum
fbr1 F. brasilicum
sa2 F. boothii
fbo2 F. boothii
fbo3 F. boothii
fbo1 F. boothii
flu1 F. louisianense
flu2 F. louisianense
fge1 F. gerlachii
fge2 F. gerlachii
us2 F. graminearum s.s.
ca2 F. graminearum s.s.
ar6 F. graminearum s.s.
un2 F. graminearum s.s.
un1 F. graminearum s.s.
us1 F. graminearum s.s.
ca1 F. graminearum s.s.
ge3 F. graminearum s.s.
ru13 F. graminearum s.s.
us3 F. graminearum s.s.
I
ar4 F. graminearum s.s. II
sy1 F. graminearum s.s. III
ar1 F. graminearum s.s. IV
ru9 F. graminearum s.s.
ru11 F. graminearum s.s.
ge11 F. graminearum s.s.
po22 F. graminearum s.s.
ne1 F. graminearum s.s.
po9 F. graminearum s.s.
fr6 F. graminearum s.s.
ge1 F. graminearum s.s.
po7 F. graminearum s.s.
ge9 F. graminearum s.s.
ru1 F. graminearum s.s.
po17 F. graminearum s.s.
ru10 F. graminearum s.s.
is1 F. graminearum s.s.
ru12 F. graminearum s.s.
ru3 F. graminearum s.s.
ge13 F. graminearum s.s.
ar3 F. graminearum s.s.
ru4 F. graminearum s.s.
bra1 F. graminearum s.s.
ar5 F. graminearum s.s.
it1 F. graminearum s.s.
it2 F. graminearum s.s.
it4 F. graminearum s.s.
it5 F. graminearum s.s.
it3 F. graminearum s.s.
it6 F. graminearum s.s.
XIII
ge12 F. graminearum s.s. XII
ge2 F. graminearum s.s.
ge5 F. graminearum s.s.
ne7 F. graminearum s.s.
ne2 F. graminearum s.s.
ge6 F. graminearum s.s.
lu8 F. graminearum s.s.
ne6 F. graminearum s.s.
fr8 F. graminearum s.s.
ge18 F. graminearum s.s.
lu1 F. graminearum s.s.
lu2 F. graminearum s.s.
ge19 F. graminearum s.s.
ge10 F. graminearum s.s.
lu5 F. graminearum s.s.
lu9 F. graminearum s.s.
fr1 F. graminearum s.s.
fr3 F. graminearum s.s.
ge15 F. graminearum s.s.
fr5 F. graminearum s.s.
ge16 F. graminearum s.s.
fr4 F. graminearum s.s.
lu6 F. graminearum s.s.
ne8 F. graminearum s.s.
ne4 F. graminearum s.s.
sa1 F. graminearum s.s.
be1 F. graminearum s.s.
lu7 F. graminearum s.s.
lu3 F. graminearum s.s.
fr7 F. graminearum s.s.
ge14 F. graminearum s.s.
fr2 F. graminearum s.s.
ge7 F. graminearum s.s.
ge4 F. graminearum s.s.
lu4 F. graminearum s.s.
XI
ne5 F. graminearum s.s.
po11 F. graminearum s.s.
X
po4 F. graminearum s.s.
po21 F. graminearum s.s.
IX
po18 F. graminearum s.s.
po13 F. graminearum s.s.
ru8 F. graminearum s.s.
po15 F. graminearum s.s.
po20 F. graminearum s.s.
po12 F. graminearum s.s.
po1 F. graminearum s.s.
po10 F. graminearum s.s.
ge17 F. graminearum s.s.
po6 F. graminearum s.s.
VIII
po23 F. graminearum s.s. VII
ir1 F. graminearum s.s.
po5 F. graminearum s.s.
po16 F. graminearum s.s.
ne3 F. graminearum s.s.
po3 F. graminearum s.s.
po14 F. graminearum s.s.
po2 F. graminearum s.s.
VI
ru7a F. graminearum s.s.
ru7 F. graminearum s.s.
V
fpse F. pseudograminearum
0.009

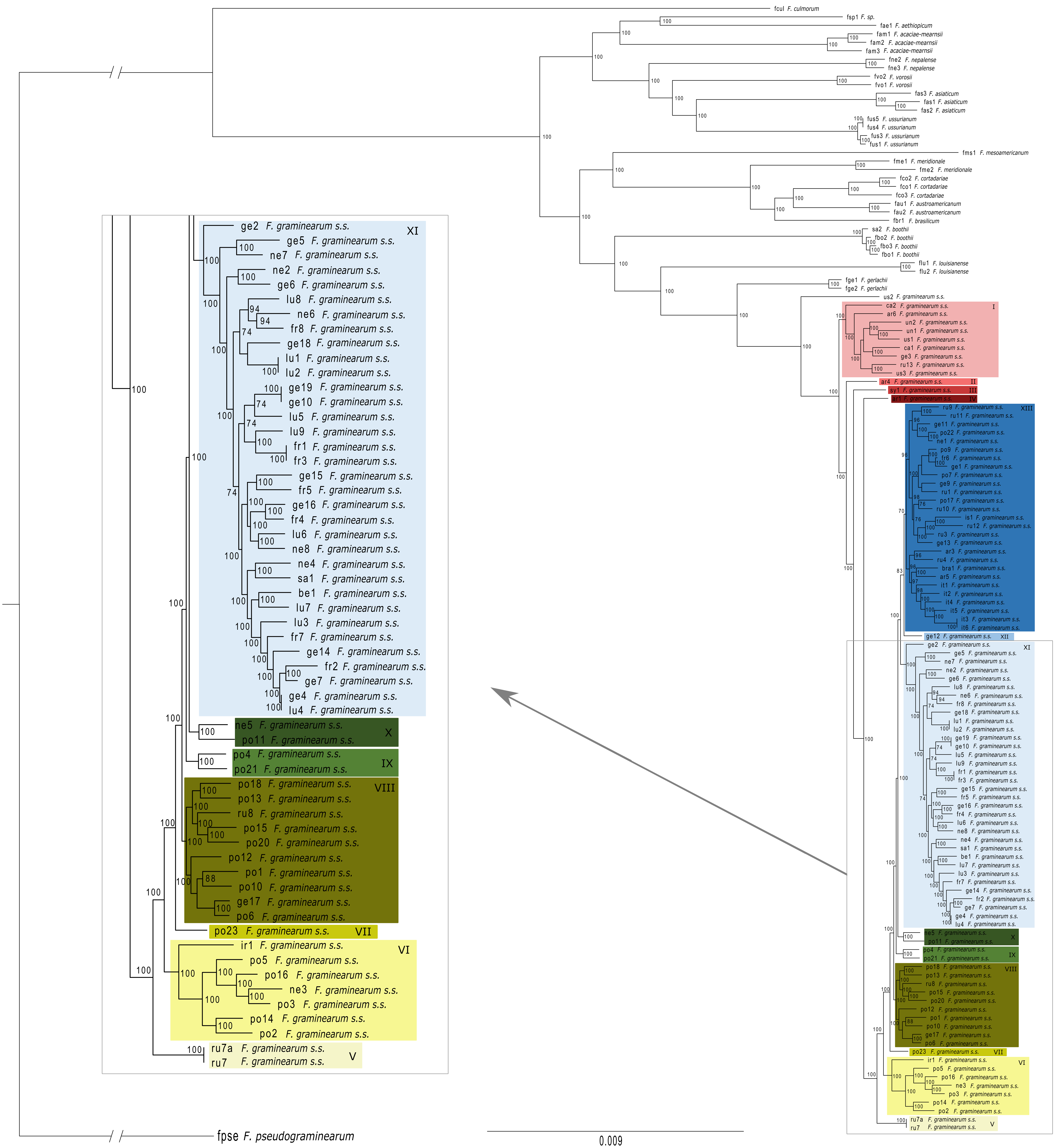

fcul F. culmorum
fsp1 F. sp.
fae1 F. aethiopicum
fam1 F. acaciae-mearnsii
fam2 F. acaciae-mearnsii
fam3 F. acaciae-mearnsii
fne2 F. nepalense
fne3 F. nepalense
fvo2 F. vorosii
fvo1 F. vorosii
fas3 F. asiaticum
fas1 F. asiaticum
fas2 F. asiaticum
fus5 F. ussurianum
fus4 F. ussurianum
fus3 F. ussurianum
fus1 F. ussurianum
fms1 F. mesoamericanum
fme1 F. meridionale
fme2 F. meridionale
fco2 F. cortadariae
fco1 F. cortadariae
fco3 F. cortadariae
fau1 F. austroamericanum
fau2 F. austroamericanum
fbr1 F. brasilicum
sa2 F. boothii
fbo2 F. boothii
fbo3 F. boothii
fbo1 F. boothii
flu1 F. louisianense
flu2 F. louisianense
fge1 F. gerlachii
fge2 F. gerlachii
us2 F. graminearum s.s.
I
ca2 F. graminearum s.s.
ar6 F. graminearum s.s.
un2 F. graminearum s.s.
un1 F. graminearum s.s.
us1 F. graminearum s.s.
ca1 F. graminearum s.s.
ge3 F. graminearum s.s.
ru13 F. graminearum s.s.
us3 F. graminearum s.s.
ar4 F. graminearum s.s. II
sy1 F. graminearum s.s. III
ar1 F. graminearum s.s. IV
XIII
ru9 F. graminearum s.s.
ru11 F. graminearum s.s.
ge11 F. graminearum s.s.
po22 F. graminearum s.s.
ne1 F. graminearum s.s.
po9 F. graminearum s.s.
fr6 F. graminearum s.s.
ge1 F. graminearum s.s.
po7 F. graminearum s.s.
ge9 F. graminearum s.s.
ru1 F. graminearum s.s.
po17 F. graminearum s.s.
ru10 F. graminearum s.s.
is1 F. graminearum s.s.
ru12 F. graminearum s.s.
ru3 F. graminearum s.s.
ge13 F. graminearum s.s.
ar3 F. graminearum s.s.
ru4 F. graminearum s.s.
bra1 F. graminearum s.s.
ar5 F. graminearum s.s.
it1 F. graminearum s.s.
it2 F. graminearum s.s.
it4 F. graminearum s.s.
it5 F. graminearum s.s.
it3 F. graminearum s.s.
it6 F. graminearum s.s.
ge12 F. graminearum s.s. XII
XI
ge2 F. graminearum s.s.
ge5 F. graminearum s.s.
ne7 F. graminearum s.s.
ne2 F. graminearum s.s.
ge6 F. graminearum s.s.
lu8 F. graminearum s.s.
ne6 F. graminearum s.s.
fr8 F. graminearum s.s.
ge18 F. graminearum s.s.
lu1 F. graminearum s.s.
lu2 F. graminearum s.s.
ge19 F. graminearum s.s.
ge10 F. graminearum s.s.
lu5 F. graminearum s.s.
lu9 F. graminearum s.s.
fr1 F. graminearum s.s.
fr3 F. graminearum s.s.
ge15 F. graminearum s.s.
fr5 F. graminearum s.s.
ge16 F. graminearum s.s.
fr4 F. graminearum s.s.
lu6 F. graminearum s.s.
ne8 F. graminearum s.s.
ne4 F. graminearum s.s.
sa1 F. graminearum s.s.
be1 F. graminearum s.s.
lu7 F. graminearum s.s.
lu3 F. graminearum s.s.
fr7 F. graminearum s.s.
ge14 F. graminearum s.s.
fr2 F. graminearum s.s.
ge7 F. graminearum s.s.
ge4 F. graminearum s.s.
lu4 F. graminearum s.s.
X
ne5 F. graminearum s.s.
po11 F. graminearum s.s.
IX
po4 F. graminearum s.s.
po21 F. graminearum s.s.
VIII
po18 F. graminearum s.s.
po13 F. graminearum s.s.
ru8 F. graminearum s.s.
po15 F. graminearum s.s.
po20 F. graminearum s.s.
po12 F. graminearum s.s.
po1 F. graminearum s.s.
po10 F. graminearum s.s.
ge17 F. graminearum s.s.
po6 F. graminearum s.s.
po23 F. graminearum s.s. VII
VI
ir1 F. graminearum s.s.
po5 F. graminearum s.s.
po16 F. graminearum s.s.
ne3 F. graminearum s.s.
po3 F. graminearum s.s.
po14 F. graminearum s.s.
po2 F. graminearum s.s.
V
ru7a F. graminearum s.s.
ru7 F. graminearum s.s.
fpse F. pseudograminearum
0.009
