# Supplementary material for: Whole-genome single nucleotide polymorphism analysis for typing the pandemic pathogen Fusarium graminearum sensu stricto
Source: Front Microbiol. 2022 Jul 18;13:885978. doi: 10.3389/fmicb.2022.885978 (PMC9339996; doi:10.3389/fmicb.2022.885978)

Supplementary Image 2. The phylogenetic tree resulting from the Maximum Likelihood on the combined alignment of 39 single-copy genes showing branches with bootstrap support values > 70%. The tree was rooted to *F. culmorum* (GenBank assembly accession: GCA_019055245.1) and *F. cerealis* (GenBank assembly accession: GCA_019055205.1)

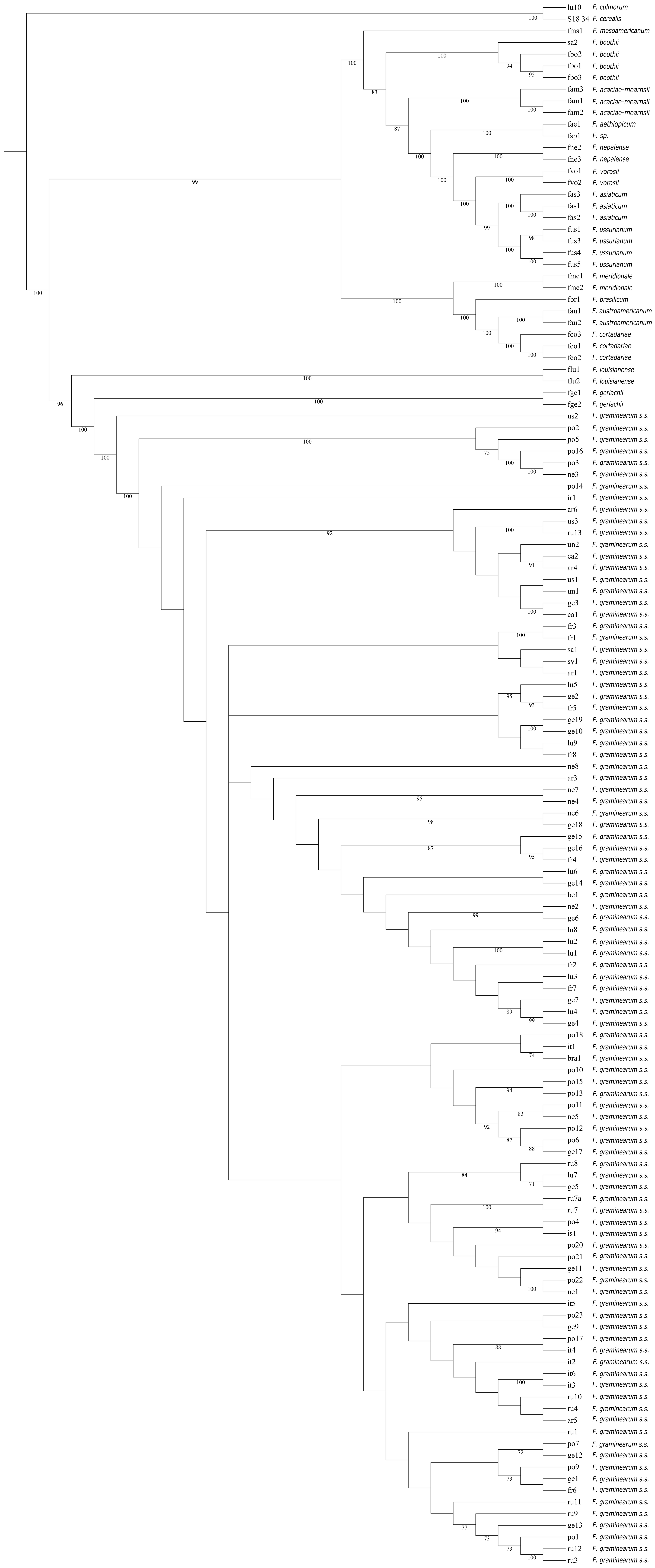

Supplement: Supplementary file 5 [file Image_2.pdf]
